# Can a Smartphone Diagnose Parkinson Disease? A Deep Neural Network Method and Telediagnosis System Implementation

**DOI:** 10.1155/2017/6209703

**Published:** 2017-09-18

**Authors:** Y. N. Zhang

**Affiliations:** ^1^School of Computer Science and Technology, Beijing Institute of Technology, Beijing 100081, China; ^2^College of Computer Science and Information Engineering, Tianjin University of Science and Technology, Tianjin 300222, China

## Abstract

Parkinson's disease (PD) is primarily diagnosed by clinical examinations, such as walking test, handwriting test, and MRI diagnostic. In this paper, we propose a machine learning based PD telediagnosis method for smartphone. Classification of PD using speech records is a challenging task owing to the fact that the classification accuracy is still lower than doctor-level. Here we demonstrate automatic classification of PD using time frequency features, stacked autoencoders (SAE), and *K* nearest neighbor (KNN) classifier. KNN classifier can produce promising classification results from useful representations which were learned by SAE. Empirical results show that the proposed method achieves better performance with all tested cases across classification tasks, demonstrating machine learning capable of classifying PD with a level of competence comparable to doctor. It concludes that a smartphone can therefore potentially provide low-cost PD diagnostic care. This paper also gives an implementation on browser/server system and reports the running time cost. Both advantages and disadvantages of the proposed telediagnosis system are discussed.

## 1. Introduction

Parkinson's disease (PD) is a disorder of brain nervous system which can cause partial or full loss in movement, behavior, and mental processing, especially speech function [1]. Generally, PD can be observed in elderly people and causes disorders in speech [2]. At present, about 1% of the worldwide population over the age of fifty is suffering from PD [3]. Until now, many effective methods and medicines [4–6] are invented for relieving the symptoms of PD. Therefore, an early-diagnosis in time and available treatment can improve the prognosis of PD [7]. However, many patients diagnosed with PD are later found which resulted in delays in patient condition [8]. Moreover, many patients with Parkinson's disease in the community still remain undiagnosed and more patients get worse because of poor medical conditions in low income areas [9].

Even though many clinical examinations and diagnostic to PD have been proposed [10–14], it is important that we should exert more effort in automated diagnosis and telediagnosis [15] in real world. In the research of Esteva et al. [16], they deduced that billions of smartphones have the potential to provide medical care for skin cancer diagnosis. Inspired by this novel idea, a smartphone also has the potential to diagnose PD. Many clinical reports reveal that the dysphonic indicator is an important reference index in diagnosis [17]. Therefore, our method is based on the study of vocal impairment symptoms (dysphonia) (90% of people with PD have such symptom) [18].

The purpose of this research is to design a machine learning based telediagnosis PD system for patients by using a smartphone. We found that deep neural network [19] with *K* nearest neighbor (KNN) [20] method can achieve better performance on available speech datasets than other comparative methods. Not only is the proposed telediagnosis system compared with other researchers' methods, but also the running time cost is tested in browser/server system. To conclude, the contribution of this paper includes (1) achieving high performance and (2) proposing a feasible implementation with empirical test.

This paper is organized as follows: in Section 2, background and related works are presented. In Section 3, the proposed method and telediagnosis system are described.  Section 4 shows the experimental results. Conclusions are drawn in Section 5.

## 2. Background and Related Works

### 2.1. Parkinson's Disease and Speech Disorders

Many researchers have exerted much effort on PD in their researches. In 2006, Rao et al. [23] discussed diagnosis and treatment for PD. The author insisted that psychosis is usually drug induced; further it can be managed initially by reducing antiparkinsonian medications. Jankovic and Aguilar [24] reviewed approaches to the treatment of PD and the authors think that the new treatments are not necessarily better than the established conventional therapy and that the treatment options must be individualized and tailored to the needs of each individual patient. In 2010, Varanese et al. [25] showed treatment of advanced PD, and the research paper concluded that supportive care, including physical and rehabilitative interventions, speech therapy, occupational therapy, and nursing care, has a key role in the late stage of disease. Yitayeh and Teshome [26] reviewed the effectiveness of physiotherapy treatment on balance dysfunction and postural instability in persons with Parkinson's disease and the author also presented meta-analysis results, in 2016.

So far, it has been reported that of the 89% of PD patients with voice and speech disorders [27, 28], the reduced speech ability to communicate is considered to be one of the most important aspects of PD by many patients [29]. The common perceptual features of reduced loudness (hypophonia), reduced pitch variation (monotone), breathy and hoarse voice quality, and imprecise articulation [30], together with lessened facial expression (masked faces), contribute to limitations in communication in the majority of people with PD [31].

**Figure 1:**
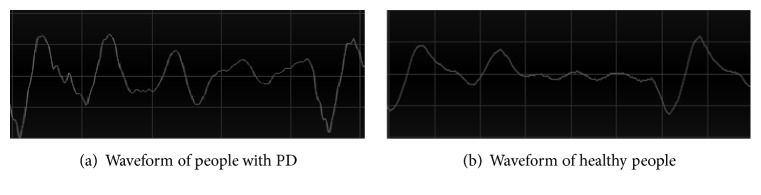
Waveform of voice records from Istanbul University [21, 22] (*x*-axis: time duration, *y*-axis: amplitude of the signal).

Figure 1 shows the comparison of waveforms between people with PD and healthy people. Intuitively, it can be observed that the waveform of healthy people is smooth and continuous, but the waveform of people with PD contains unexpected vibrations. The reason for this phenomenon is that the people with PD lose the ability of precise muscle control [32–34]. The vibrations can be detected by time frequency analysis; moreover machine learning based audio analysis methods are appropriate for diagnosing PD.

### 2.2. Machine Learning for Parkinson's Disease Diagnostic Care

Many researchers had proposed effective methods based on machine learning in automated diagnosis research. In 2014, Shahbakhi et al. [43] proposed a method using genetic algorithm and support vector machine for analysis of speech for diagnosis of PD. Little et al. [44] used support vector machine (SVM) with Gaussian radical basis kernel to diagnose PD. Shahbaba and Neal [35] showed a nonlinear model for the PD classification which is based on Dirichlet mixtures. Sakar and Kursun [45] applied mutual information measure to combine with SVM. Those methods achieve high classification accuracy but telediagnosis PD needs a better method with higher classification performance.

Recently, deep neural networks have shown potentials in speech recognition tasks; the classification and recognition accuracy is superior to conventional machine learning method. We proposed a method using deep neural network (stacked autoencoders, SAE) to reduce dimensions and *K* nearest neighbor classifier to diagnose PD. We also implemented a telediagnosis system based on the proposed method. Results of empirical test on smartphone are presented in Section 4.

## 3. Methodology

### 3.1. Structure of PD Telediagnosis Method and System Structure

**Figure 2:**
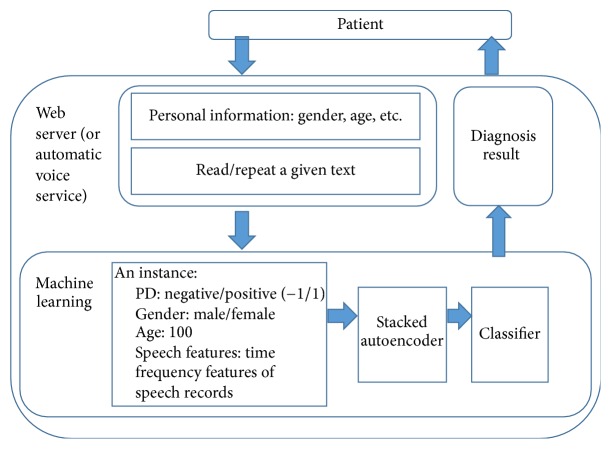
Acquisition and diagnosis of PD telediagnosis method.

The proposed structure of the PD telediagnosis method is as shown in Figure 2. A patient provides personal information and speech records by following instructions of smartphone. The personal information includes gender, age, and a brief health history. Patient is also asked to read a given text; then the speech records of the patient are parsed to be time frequency based features which are extracted from the voice samples. After the processing of SAE and KNN, patient can receive the diagnosis result.

**Figure 3:**
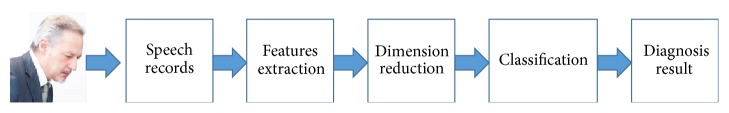
Workflow of the proposed method.

Figure 3 shows the workflow of the proposed method in the view of machine learning, an appropriate set of time frequency features, SAE, and classifier dictated diagnostic accuracy. Therefore, the most important work is how to build a high accuracy diagnostic method.

**Figure 4:**
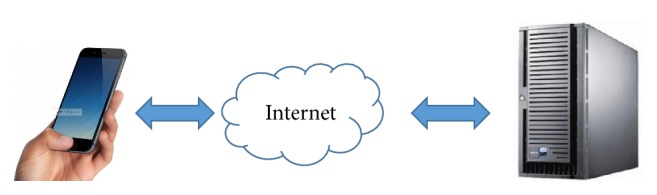
B/S (browser/server) structure.

Figure 4 briefly illustrates the architecture of the proposed method on B/S (browser/server) structure and details are shown in Section 4.4. The server should be installed on an operation system. In next step, an appropriate version of web service software should be deployed on this server. Usually a smartphone embedded Internet browser software (such as Google Chrome App). Therefore the smartphone can send/receive data (text and voice records) to server by Internet browser. The connection between smartphone and server can be 2/3/4G mobile network or WIFI. The server receives and processes audio files as Figures 1 and 2 present. Result of PD diagnosis will be displayed on patient's smartphone. This B/S structure is not limited to smartphone, but it is suitable for other electronic devices too, such as iPad, notebook, and even a smart watch. Moreover, the automated voice service system (such as banks' telephone services) should fulfill the same task as B/S structure theoretically.

### 3.2. Speech Features

Dysphonia [17] is a typical speech problem of people with PD. Dysphonia is a human vocal problem which includes five major clinical features: loudness, decrease, breathiness, roughness, and exaggerated vocal tremor in voice. All those indications can be detected by analyzing time frequency in speech records. A set of 26 features is listed with the considering of the previous works held on this field of study [21, 46].

In Table 1, 6 types of parameters are listed; they are frequency parameters, pulse parameters, amplitude parameters, voicing parameters, pitch parameters, and harmonicity parameters. 26 features are also presented in Table 1.

### 3.3. Stacked Autoencoders

**Figure 5:**
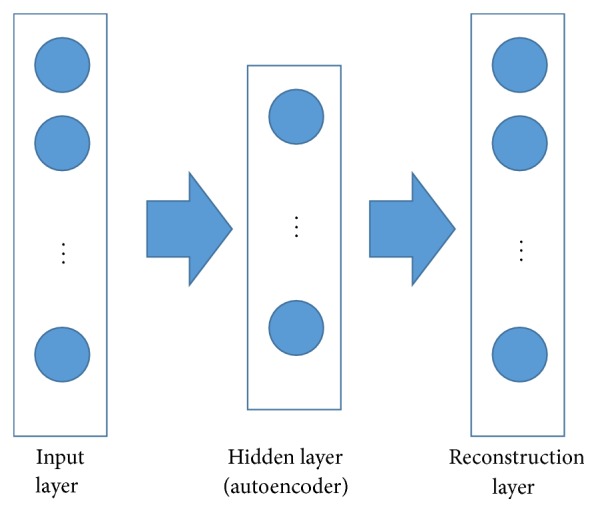
Autoencoder.

Autoencoder [47] has been wildly used in unsupervised feature learning and speech recognition tasks. It can be built as a special three-layer neural network: the input layer, the hidden layer, and the reconstruction layer (as shown in Figure 5).

An autoencoder has two parts: (1) The encoder receives an input *x*_0_ ∈ *R*^*d*0^ to the hidden layer (latent representation feature) *x*_1_ ∈ *R*^*d*1^ via a mapping *f*_encoder_:

(1) $$\begin{array}{c} x_{1}=f_{encoder}\left(\;x_{0}\;\right)=s_{encoder}\left(\;W_{1}^{T}x_{0}+b_{1}\;\right), \end{array}$$

where *s*_encoder_ is the activation function, whose input is called the activation of the hidden layer, and {*W*_1_, *b*_1_} is the parameter set with a weight matrix *W*_1_ ∈ *R*^*d*0*∗d*1^ and a bias value vector *b*_1_ ∈ *R*^*d*1^.

(2) The decoder maps the hidden representation *x*_1_ back to reconstruction *x*_2_ ∈ *R*^*d*0^ via mapping function *f*_decoder_:

(2) $$\begin{array}{c} x_{2}=f_{decoder}\left(\;x_{1}\;\right)=s_{decoder}\left(\;W_{2}^{T}x_{1}+b_{2}\;\right). \end{array}$$

*s*_decoder_ is the activation function of the decoder with parameters {*W*_2_, *b*_2_}, *W*_2_ ∈ *R*^*d*1*∗d*0^, *b*_2_ ∈ *R*^*d*0^. The input of *s*_decoder_ is defined as the activation of the reconstruction layer. Parameters are learned through backpropagation by minimizing the loss *L*(*x*_0_, *x*_2_):

(3) $$\begin{array}{c} L\left(\;x_{0},x_{2}\;\right)=L_{r}\left(\;x_{0},x_{2}\;\right)+0.5\varepsilon \left(\;{\left\|\;W_{1}\;\right\|}_{2}^{2}+{\left\|\;W_{2}\;\right\|}_{2}^{2}\;\right) \end{array}$$

In (3), *L*(*x*_0_, *x*_2_) consists of the reconstruction error *L*_*r*_ and the *L*_2_ regularization of *W*_1_ and *W*_2_. By minimizing the reconstruction error *L*_*r*_, the hidden feature should be able to reconstruct the original input *x*_0_ as much as possible.

**Figure 6:**
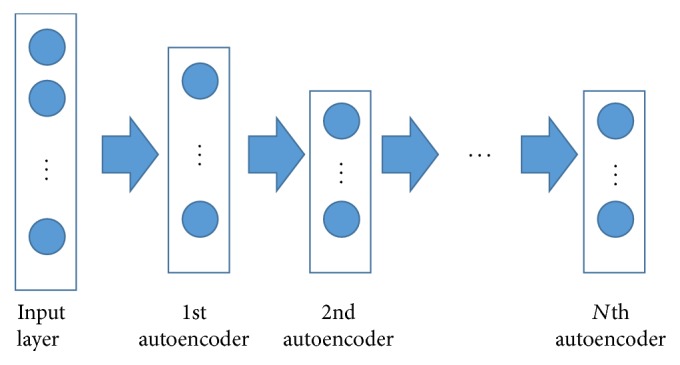
Stacked autoencoders.

The stacked autoencoders (SAE) [48] are multiple layers of autoencoders. They are a deep learning approach for dimensionality reduction and feature learning. As Figure 6 shows, there are n autoencoders which are trained one by one. The input vectors are fed to the first autoencoder. After finishing training the first autoencoder, the output hidden representation is propagated to the second auto layer. A typical activation function is sigmoid function which is used for activation functions *s*_encoder_ and *s*_decoder_. After this pretraining stage, the whole SAE is fine-tuned [48] based on a predefined objective. The last hidden layer of the SAE encoder can further cooperate with other applications, such as SVM for classification task.

### 3.4. KNN Classifier

The KNN [49] classifier is quite simple: given a speech record of undiagnosed patient *s*, the system finds the *K* nearest neighbors to give diagnosis result. Formally, the decision rule can be written as

(4) $$\begin{array}{c} score\left(\;{s,c}_{i}\;\right)=\sum_{S_{j}\in KNN\left(S\right)}Sum\left(\;{S,S}_{j}\;\right)\delta \left(\;{S_{j},c}_{i}\;\right). \end{array}$$

Above, KNN(*S*) indicates the set of *K* nearest neighbors of speech records *S*. *δ*(*S*, *c*_*i*_) is the classification for undiagnosed patient *s* with respect to class *c*_*i*_, and

(5) $$\begin{array}{c} \delta \left(\;{s,c}_{i}\;\right)=\left\{\begin{array}{cc} 1 & s\in c_{i}\\ -1 & s\notin c_{i}. \end{array}\right. \end{array}$$

For undiagnosed patient, the patient could be given diagnosis result. *K* of KNN is 1 in this paper.

## 4. Experimental Results

### 4.1. Speech Datasets, Evaluation Criteria, and Classifiers

For comparison, we chose two research papers [21, 50] and the PD speech datasets they had used. We use Matlab as programming tool and all classification algorithms are determined by grid search method.

In the first paper, Sakar et al. [21] collected a speech dataset of diagnosis of PD and donated this speech dataset to UCI machine learning group for other researchers. This speech dataset contains a training set file, a testing set file, and a ZIP package of WMA files. Betul Erdogdu Sakar et al. designed a novel speech test; PD patients were asked to say only the sustained vowels “a” and “o” three times. Their work of speech dataset collection was finished at Istanbul University and we call the dataset “Istanbul Dataset” in the following experiments.

In the second paper, Ma et al. [50] proposed a kernel extreme learning machine with subtractive clustering features weighting approach. Their method compared with 15 researches' methods. Total 16 methods are compared in their research paper, and the method of Ma et al. gained top position. The dataset of their research was created by Little et al. [44] of the University of Oxford, in collaboration with the National Centre for Voice and Speech, Denver, Colorado, who recorded the speech signals. We call the dataset “Oxford Dataset” in the following experiments.

Accuracy, sensitivity, and other performance indexes are compared among those classifiers and these performance indexes are defined as follows:

(6) $$\begin{array}{c} Accuracy=\frac{TP+TN}{TP+FP+TN+FN}. \end{array}$$

Sensitivity:
  (7) $$\begin{array}{c} Sensitivity=\frac{TP}{P}=\frac{TP}{TP+FN}. \end{array}$$
Specificity:
  (8) $$\begin{array}{c} specificity=\frac{TN}{N}=\frac{TN}{FP+TN}. \end{array}$$
*F*-score:
  (9) $$\begin{array}{c} F\text{-score}=\frac{2TP}{2TP+FP+FN}. \end{array}$$
MCC:
  (10) MCC=TP∗TN−FP∗FNTP+FP∗TP+FN∗TN+FP∗TN+FN,

where TP, FP, TN, and FN are the true and false positive and true and false negative classifications of a classifier and they are defined as shown in Table 2.

Involved classifiers are kernel extreme learning machine (KELM), Linear Support Vector Machine (LSVM), Multilayer perceptrons Support Vector Machine (MSVM), Radial basis function Support Vector Machine (RSVM), Classification and Regression Tree (CART), KNN, Linear Discriminant Analysis (LDA), and Naive Bayesian (NB) method.

### 4.2. Experiment Results: Oxford Dataset and Algorithm Parameters

The Oxford Dataset [22, 46] consisted of voice measurements from 31 people and 23 of them were diagnosed with PD. There are total 195 samples comparing 147 PD and 48 healthy samples in the dataset. There are no missing values in the Oxford Dataset and each feature is real type value. The whole 22 features are contained in Table 1.

The SAE of the proposed method has two hidden layer: layer 1 and layer 2. The size of layer 1 is set as 10, 9, or 8 neurons and the size of layer 2 is set as 8, 7, or 6 neurons. The batch size of SAE is 20 in training and fine tuning step. All comparative classifiers are optimized by grid search method. We use 50-50% training-testing method as Polat [40] and Daliri [39] did. This experiment method tests comparative approaches with much less training data than 10-fold cross validation. Each classifier was tested 10 times and the results are presented in tables.

#### 4.2.1. Classifiers without Deep Neural Network on Oxford Dataset

Table 3 summarized the detailed results of classification accuracy after 10 runs. From this table, it can be found that the classification performance of KNN is apparently differential. We can see that the KNN outperforms that with other 7 classifiers with a max, mean, and min accuracy of 90.53%, 82.76%, and 76.84%. All comparative classifiers give low classification results because the input samples are 22-dimensional data.

#### 4.2.2. Classifiers with Deep Neural Network on Oxford Dataset

Tables 4 and 5 presented the comparison result of the classification accuracy and other performance indexes.  Table 4 lists details of accuracy. For SAE, the influence of two layers in dimension reduction has been investigated. In this study, two hidden layers are tested as a subgrid search. As seen from Table 4, 1NN classifier gives more correct diagnosis results than other methods.

Comparing Tables 3 and 4, other 7 classifiers gave more correct classification result with the using of SAE (hidden layer 1 contains 9 neurons; hidden layer 2 contains 7 neutrons). KELM has obtained max classification accuracy as 98.81%; it is 16% higher than 82.23% in Table 3. LSVM gives 96.71% classification accuracy after dimensional reduction by SAE 8-7. In SAE 10-7 row, MSVM increased 11% in terms of classification accuracy. RSVM gives 97.75% classification accuracy in the row of SAE 9-7. Similarly, CART produced average classification accuracy as 96% when applying SAE. LDA and NB also have got better performance than without using SAE; the classification accuracy of those 7 classifiers are improved by 10–15% with the using of SAE.

### 4.3. Experiment Results: Istanbul Dataset and Parameters Settings

The Istanbul University built a PD database. This database consists of training and test files. The training data belongs to 20 people with PD (6 females, 14 males) and 20 healthy individuals (10 females, 10 males) who participate in the PD database project at the Department of Neurology in Cerrahpasa Faculty of Medicine, Istanbul University. From all subjects, multiple types of sound recordings (26 voice samples including sustained vowels, numbers, words, and short sentences) are taken. A group of 26 linear and time frequency based features are extracted from each voice sample. Those features are presented in Section 3.2, as Table 2 shows.

28 PD patients are asked to say only the sustained vowels “a” and “o” three times, respectively, which makes a total of 168 recordings. The same 26 features are extracted from voice samples of this dataset. The researchers of the Istanbul University declared that [22] the PD dataset can be used as an independent test set to validate the results obtained on training set. The training file contains 1040 recordings and the testing file contains 168 files. Therefore, we used the training file and testing file to compare the proposed method with other methods.

The SAE of the proposed method has two hidden layer: layer 1 and layer 2. Layer 1 is set as 10, 9, or 8 neurons and layer 2 is set as 8, 7, or 6 neurons. The batch size of SAE is 20 in training and fine tuning step.

#### 4.3.1. Classifiers without Deep Neural Network on Istanbul Dataset

Table 6 summarized the results of classification accuracy. According to the declaration of Istanbul University, fixed training set and testing set are given. Only one run will obtain final result. From this table, it can be found that the classification performance of RSVM is apparently differential. We can see that the RSVM outperforms that with other 7 classifiers with a classification accuracy of 76.41%. All comparative classifiers give low classification results because the input samples are 26-dimensional data. The Naive Bayesian method gives worst result among all classifiers.

#### 4.3.2. Classifiers with Deep Neural Network on Istanbul Dataset

Tables 7 and 8 presented the comparison result of the classification accuracy and other 4 performance indexes.  Table 7 lists details of classification accuracy. To SAE, two hidden layers are set as Oxford Dataset experiment. As seen from Table 6, KNN classifier gives more correct diagnosis results than other methods. The max, mean, and min classification accuracy of KNN classifier are not affected by network structure of SAE.

Comparing Tables 6 and 5, the other 7 classifiers gave more correct classification result with the using of SAE. And LSVM, RSVM, and CART show much more stability in classification accuracy. But KELM still did not obtain 100.00% classification accuracy, Table 6. LSVM gives 92.00% classification accuracy after dimensional reduction by SAE 9-7. In SAE 10-7, SAE10-6, and SAE 9-6 row, LSVM did not give perfect performance. MSVM, LDA, and NB increased classification accuracy but still lack stability. In conclusion, every classifier has got better performance than without using SAE.

### 4.4. Implementation on B/S System

**Figure 7:**
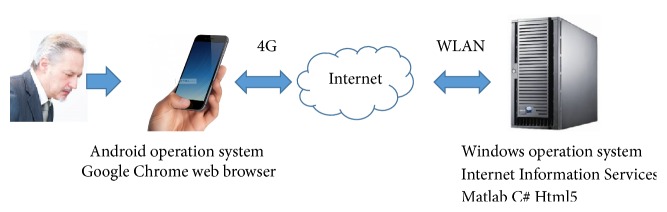
Implementation of the proposed system.

As Figure 7 shows, our smartphone is running Android system and it was installed from Google Chrome web browser. The server installed Windows operation system and Internet Information Services. We also installed Matlab 2016a and Visual Studio 2013 on this server. Programming techniques contain Matlab, C#, and HTML5.

We used Chrome to connect server via 4G mobile network in the street; we send 28 test speech records (WMA files) of Istanbul Dataset; a transmission of 12.1 MB data took less than 5 seconds to our laboratory. The server received speech records and ran the empirical experiments as Sections 4.2 and 4.3 present. No more than 2 minutes, results of all comparative classifiers were displayed on our smartphone. We also recorded our speech (as healthy samples) and send it to server for testing, but it should be noticed that a quite environment is necessary. It is hard to achieve satisfied classification accuracy in a noisy circumstance.

### 4.5. Discussions and Future Work

Table 9 summarized the comparative results achieved from related researches. It can be seen that the proposed method achieves better results than other methods and our method shows a relatively fewer training samples. The reduced training dataset is meaningful when applying the proposed method in reality. The performance of SAE and KNN is robust to number of hidden neutrons of deep neural network.

We focus on classification accuracy, sensitivity, and other performance indexes to evaluate machine learning based PD diagnosis method; then we choose a B/S structure to test the proposed architecture. Advantages contain time saving, being convenient, and low cost. But we did not achieve 100% correct classification accuracy on smartphone. We deduced that there are still potential problems: (1) a good enough microphone should be taken into consideration. (2) Speech denoising may be fulfilled by multiple microphones. (3) An evolutionary system can overcome big testing data in real world. The future investigation will pay much attention on an evolutionary telediagnosis PD system and solve above issues. Moreover, we also noticed that two public datasets cannot guarantee that a machine learning based telediagnosis system can be trained very well and it is hard to satisfy hospitals, clinics, and other medical institutions. And the stability and robustness of a telediagnosis system still need to be built urgently.

## 5. Conclusion

For building a convenient and feasible telediagnosis PD service via smartphone, we proposed a machine learning based method on browser/server architecture. In this paper, the proposed method contains stacked autoencoders and KNN classifier which is used to process speech records. The proposed method can remap time frequency features in low dimensional space. Results show that the proposed method with KNN classifier can give doctor-level classification results on public PD speech records. An experimental system is also built for testing; it is projected that telediagnosis of PD on a smartphone will be in the future.

## Figures and Tables

**Table 1:** Time frequency features.

Parameter type	Features
Frequency	Jitter (local)
Parameters	Jitter (rap)
(Number of features: 5)	Jitter (local, absolute)
	Jitter (ppq5) Jitter (ddp)

Harmonicity	Autocorrelation
Parameters	Noise-to-harmonic
(Number of features: 3)	Harmonic-to-noise

Pulse	Number of pulses
Parameters	Mean period
(Number of features: 4)	Number of periods
	Standard dev. of period

Amplitude	Shimmer (local)
Parameters	Shimmer (apq3)
(Number of features: 6)	Shimmer (local, dB)
	Shimmer (apq5)
	Shimmer (dda)
	Shimmer (apq11)

Pitch	Median pitch
Parameters	Mean pitch
(Number of features: 5)	Minimum pitch
	Maximum pitch
	Standard deviations

Voicing	Fraction of locally
Parameters	unvoiced frames
(Number of features: 4)	Number of voice breaks
	Degree of voice breaks

Types: 6	Total features: 26

**Table 2:** Confusion matrix.

	Prediction as people with PD	Prediction as healthy people
Actual people with PD	True positive (TP)	False negative (FN)
Actual healthy people	False positive (FP)	True negative (TN)

**Table 3:** Results of comparative classifiers without SAE on Oxford Dataset.

Classifier	Classification accuracy (%)	Max	Mean	Min
KELM	ACC	83.23	71.32	68.49
LSVM	ACC	81.05	64.21	44.21
MSVM	ACC	84.21	61.98	43.16
RSVM	ACC	85.26	74.34	69.47
CART	ACC	89.47	73.95	58.95
KNN	ACC	**90.53**	**82.76**	**76.84**
LDA	ACC	87.37	69.61	53.68
NB	ACC	75.79	69.74	61.05

**Table 4:** Results of comparative classifiers with SAE on Oxford Dataset.

Classification accuracy (%)		Classifiers							
		KELM	LSVM	MSVM	RSVM	CART	KNN	LDA	NB
SAE 10-8	Max	93.45	99.99	99.90	99.90	99.90	100.00	99.69	99.19
	Mean	77.74	95.89	94.47	96.32	96.04	**97.81**	95.17	94.29
	Min	66.67	93.66	89.23	93.43	93.81	93.23	92.85	93.73

SAE 10-7	Max	96.43	100.00	98.46	100.00	100.00	100.00	99.60	99.58
	Mean	80.18	96.20	95.76	96.58	96.82	**97.63**	96.16	95.22
	Min	63.69	94.13	93.46	93.52	94.53	94.52	93.01	93.61

SAE 10-6	Max	89.29	100.00	99.81	99.99	100.00	100.00	99.73	99.15
	Mean	71.73	96.26	94.74	96.51	96.43	**96.97**	94.59	95.18
	Min	45.83	93.74	89.13	93.51	94.64	93.22	91.37	93.62

SAE 9-8	Max	90.48	100.00	97.40	100.00	99.99	100.00	99.70	99.71
	Mean	78.27	95.87	95.04	96.87	96.87	**97.89**	96.08	95.12
	Min	56.55	92.36	89.04	93.99	94.24	92.70	92.71	93.54

SAE 9-7	Max	93.45	100.00	98.17	100.00	100.00	100.00	99.74	98.74
	Mean	80.77	96.23	93.88	**97.75**	96.82	97.13	97.01	95.82
	Min	65.48	94.22	89.04	93.59	94.49	93.14	93.28	93.93

SAE 9-6	Max	98.81	100.00	99.71	100.00	100.00	100.00	99.66	99.36
	Mean	84.59	96.22	95.63	96.46	96.01	**98.01**	94.03	95.51
	Min	70.83	93.31	90.58	94.16	94.30	93.26	93.55	93.94

SAE 8-8	Max	93.45	100.00	99.52	99.99	100.00	100.00	99.58	99.51
	Mean	76.61	96.02	95.39	95.64	96.22	**96.98**	95.45	94.32
	Min	55.95	94.19	92.21	93.37	94.36	92.75	93.75	92.25

SAE 8-7	Max	98.81	100.00	99.62	100.00	100.00	100.00	99.76	99.79
	Mean	84.23	96.71	95.48	96.82	96.23	96.63	95.69	**97.07**
	Min	70.83	94.40	92.21	93.92	94.57	94.10	92.99	94.14

SAE 8-6	Max	91.07	100.00	99.42	99.99	99.99	100.00	99.78	99.37
	Mean	78.22	96.01	94.52	96.21	95.91	**96.92**	95.57	95.98
	Min	57.74	93.37	90.87	93.59	92.53	93.42	93.44	92.49

**Table 5:** Performance indexes of comparative classifiers with SAE on Oxford Dataset *(average of 10 runs)*.

Performance indexes		Classifiers							
		KELM	LSVM	MSVM	RSVM	CART	KNN	LDA	NB
SAE 10-8	Specificity	0.2083	0.0889	0.0816	0.8148	0.7302	**0.9565**	0.4872	0.0286
	Sensitivity	0.0423	0.7	0.2174	0.3824	0.0625	**0.9306**	0.1429	0.2
	*F*-score	0.0645	0.5556	0.198	0.5253	0.0784	**0.9571**	0.1905	0.102
	MCC	−0.7719	−0.2635	−0.7095	0.1897	−0.245	**0.8425**	−0.3992	−0.8053

SAE 10-7	Specificity	0.3455	0.2794	0.3421	0.3256	0.7273	**0.9710**	0.6667	0.8571
	Sensitivity	0.775	0.7407	0.7193	0.7692	0.5616	**0.9231**	0.5581	0.7778
	*F*-score	0.5794	0.4167	0.6667	0.6612	0.6833	**0.9231**	0.7007	0.6512
	MCC	0.1304	0.0204	0.0653	0.1058	0.2438	**0.8941**	0.132	0.565

SAE 10-6	Specificity	0.5714	0.3387	0.7273	0.2581	**0.8621**	0.7432	0.0556	0.8806
	Sensitivity	0.5672	0.9091	0.7581	0.9375	0.3939	0.9048	0.0779	**0.9286**
	*F*-score	0.6496	0.5769	0.7966	0.8163	0.5417	0.6441	0.1200	**0.8387**
	MCC	0.1266	0.2715	0.4698	0.276	0.2536	0.5489	−0.7927	**0.7696**

SAE 9-8	Specificity	0.4462	0.2000	0.0274	0.1207	0.4615	**0.6066**	0.2500	0.6316
	Sensitivity	0.8333	**0.9091**	0.3182	0.3243	0.1829	0.8235	0.2069	0.7368
	*F*-score	0.5495	0.7299	0.1400	0.2400	0.2885	**0.6512**	0.3243	0.6437
	MCC	0.271	0.1567	−0.7202	−0.5726	−0.2897	**0.4142**	−0.3471	0.3612

SAE 9-7	Specificity	0.2597	0.125	0.0455	0.3667	0.4000	**0.9492**	0.4792	0.2917
	Sensitivity	0.0556	0.4366	0.2192	0.2857	0.6222	0.5000	0.2766	**0.7447**
	*F*-score	0.0263	0.5041	0.2909	0.2410	**0.7517**	0.6316	0.3059	0.6034
	MCC	−0.5503	−0.3827	−0.6362	−0.3354	0.0102	**0.5251**	−0.2493	0.0408

SAE 9-6	Specificity	0.8929	0.8462	0.0606	0.7857	0.3404	**0.9500**	0.0879	0.9241
	Sensitivity	0.7612	0.2558	0.0484	0.0256	0.7500	**0.8182**	0.2500	0.5000
	*F*-score	0.8430	0.3548	0.0625	0.0385	0.6261	**0.8824**	0.0227	0.5333
	MCC	0.6021	0.1269	−0.8850	−0.2700	0.0992	**0.7586**	−0.4156	0.4477

SAE 8-8	Specificity	0.1522	**1.0000**	0.9286	0.2683	0.8409	0.9889	0.6111	0.3542
	Sensitivity	0.1429	0.7647	0.5373	0.6111	0.5490	0.6000	**0.9870**	0.234
	*F*-score	0.1474	0.8667	0.6857	0.5641	0.6512	0.6667	**0.9500**	0.2472
	MCC	−0.705	**0.8529**	0.4336	−0.1264	0.4031	0.6548	0.7056	−0.4146

SAE 8-7	Specificity	0.7222	0.7500	0.3529	0.7500	0.1600	**0.9808**	0.2188	0.4118
	Sensitivity	**0.7805**	0.1205	0.0769	0.011	0.3143	0.7442	0.1290	0.4103
	*F*-score	0.7273	0.2083	0.1263	0.0215	0.3894	**0.8421**	0.0941	0.5333
	MCC	0.4980	−0.1252	−0.5701	−0.3344	−0.4651	**0.7579**	−0.6174	−0.1374

SAE 8-6	Specificity	0.8462	0.1333	0.375	0.125	0.2414	0.0308	**0.9231**	0.557
	Sensitivity	0.3415	0.6000	0.5057	0.7419	0.3333	0.1667	**0.7857**	0.6875
	*F*-score	0.5000	0.6809	0.6471	0.4182	0.4000	0.1020	**0.8544**	0.3548
	MCC	0.1387	−0.2028	−0.0663	−0.1667	−0.3928	−0.8271	**0.6974**	0.1831

**Table 6:** Results of comparative classifiers without SAE on Istanbul Dataset.

Classifier	Classification accuracy (%)	Value
KELM	ACC	57.83
L-SVM	ACC	39.88
M-SVM	ACC	75.60
R-SVM	ACC	**76.41**
CART	ACC	50.00
KNN	ACC	55.95
LDA	ACC	57.28
NB	ACC	30.95

**Table 7:** Results of comparative classifiers with SAE on Istanbul Dataset.

Classification accuracy (%)		Classifiers							
		KELM	LSVM	MSVM	RSVM	CART	KNN	LDA	NB
SAE 10-8	Max	93.45	100.00	99.90	100.00	100.00	100.00	100.00	100.00
	Mean	77.74	67.83	88.03	79.90	91.58	93.45	83.93	81.97
	Min	66.67	57.08	77.20	74.24	71.74	89.23	75.95	80.31

SAE 10-7	Max	96.43	100.00	98.46	100.00	100.00	100.00	100.00	100.00
	Mean	80.18	67.73	88.24	80.48	91.25	93.99	83.88	82.59
	Min	63.69	56.34	76.80	74.47	72.31	89.59	76.37	79.80

SAE 10-6	Max	89.29	100.00	99.81	100.00	100.00	100.00	100.00	99.90
	Mean	71.73	67.36	88.18	80.65	91.73	93.72	84.70	82.09
	Min	45.83	56.79	77.01	73.72	72.45	90.06	76.60	79.70

SAE 9-8	Max	90.48	100.00	97.40	100.00	100.00	100.00	99.81	100.00
	Mean	78.27	67.90	88.63	80.57	91.55	93.98	84.39	82.29
	Min	56.55	56.87	77.10	73.92	72.23	89.82	76.32	80.27

SAE 9-7	Max	93.45	100.00	98.17	100.00	100.00	100.00	99.90	99.71
	Mean	80.77	67.85	88.67	79.76	91.19	94.17	84.35	81.88
	Min	65.48	56.45	76.98	74.04	72.07	90.21	76.11	79.73

SAE 9-6	Max	98.81	100.00	99.71	100.00	100.00	100.00	100.00	100.00
	Mean	84.59	67.67	88.50	80.36	91.17	93.78	84.69	82.02
	Min	70.83	57.01	77.62	74.04	71.94	90.13	75.89	79.68

SAE 8-8	Max	93.45	100.00	99.52	100.00	100.00	100.00	99.81	100.00
	Mean	76.61	67.96	88.47	80.08	91.14	94.10	84.23	81.89
	Min	55.95	57.24	76.72	74.21	72.29	89.31	75.76	80.40

SAE 8-7	Max	98.81	100.00	99.62	100.00	100.00	100.00	100.00	100.00
	Mean	84.23	68.27	87.92	80.34	91.56	94.35	84.55	82.08
	Min	70.83	57.12	77.17	73.95	72.61	89.87	76.26	79.74

SAE 8-6	Max	91.07	100.00	99.42	100.00	100.00	100.00	100.00	100.00
	Mean	78.22	67.49	87.85	79.89	91.01	93.81	84.24	81.75
	Min	57.74	56.62	76.69	73.82	71.88	90.05	75.80	80.05

**Table 8:** Performance indexes of comparative classifiers without SAE on Istanbul Dataset (average values of 10 runs).

Performance indexes		Classifiers							
		KELM	LSVM	MSVM	RSVM	CART	KNN	LDA	NB
SAE 10-8	Specificity	0.4474	0.7445	0.6667	0.8429	0.3684	0.8000	0.8904	0.8807
	Sensitivity	0.8692	0.3548	0.9149	0.5714	0.9799	0.9412	0.8000	0.6949
	*F*-score	0.8561	0.2857	0.9247	0.4848	0.9511	0.9600	0.8492	0.7257
	MCC	0.3297	0.0864	0.5577	0.369	0.4662	0.6391	0.6845	0.5884

SAE 10-7	Specificity	0.9551	0.9259	0.8046	0.5263	0.9231	0.9537	0.886	0.8193
	Sensitivity	0.6203	0.4368	0.963	0.8389	0.9052	0.9000	0.7222	0.8235
	*F*-score	0.7424	0.5802	0.8864	0.8834	0.9333	0.9076	0.7358	0.8235
	MCC	0.6179	0.4122	0.7737	0.2879	0.8021	0.8569	0.6144	0.6428

SAE 10-6	Specificity	0.7958	0.2545	0.9714	0.8397	0.9593	0.9275	0.8875	0.8293
	Sensitivity	0.2692	0.8761	0.7302	0.3333	0.800	0.9394	0.8068	0.7778
	*F*-score	0.2258	0.7826	0.8214	0.1951	0.8372	0.9442	0.8452	0.6931
	MCC	0.0573	0.1645	0.7473	0.1179	0.7829	0.8651	0.6943	0.5703

SAE 9-8	Specificity	0.9848	0.6842	0.9107	0.0909	0.9595	0.9375	0.9778	0.9067
	Sensitivity	0.0278	0.6712	0.8661	0.9110	0.5500	0.9318	0.7886	0.1111
	*F*-score	0.0513	0.6447	0.9065	0.8896	0.5946	0.9371	0.8778	0.1176
	MCC	0.0391	0.3530	0.7498	0.0022	0.5471	0.8689	0.6884	0.0187

SAE 9-7	Specificity	0.8874	0.5326	0.9063	0.8584	0.9478	0.9263	0.783	0.4706
	Sensitivity	0.0588	0.8421	0.8750	0.6545	0.7647	0.9589	0.9355	0.8543
	*F*-score	0.0571	0.6995	0.9225	0.6729	0.7761	0.9333	0.8112	0.8927
	MCC	−0.0524	0.3878	0.688	0.5207	0.7205	0.8807	0.6939	0.2558

SAE 9-6	Specificity	0.2778	0.7050	0.9764	0.7265	0.9833	0.9469	0.8257	1
	Sensitivity	0.9133	0.5172	0.5854	0.9804	0.8704	0.9091	0.8814	0.6517
	*F*-score	0.9133	0.3529	0.7059	0.7519	0.9261	0.9009	0.8000	0.7891
	MCC	0.1911	0.1782	0.657	0.6502	0.8252	0.8521	0.6832	0.6841

SAE 8-8	Specificity	0.7956	0.6964	0.8909	0.8500	0.65	0.9845	0.5714	0.7538
	Sensitivity	0.6129	0.6429	0.3333	0.7813	0.9459	0.7949	0.9098	0.8544
	*F*-score	0.4872	0.5714	0.0909	0.8547	0.9492	0.8611	0.8996	0.8502
	MCC	0.3530	0.3244	0.0938	0.5572	0.5836	0.8282	0.4977	0.6100

SAE 8-7	Specificity	0.7830	0.8455	0.7273	0.947	0.9292	0.9130	0.6792	0.9766
	Sensitivity	0.9355	0.2222	0.9111	0.2500	0.8727	0.9508	0.9217	0.3000
	*F*-score	0.8112	0.2703	0.9213	0.3462	0.8649	0.9587	0.8908	0.4364
	MCC	0.6939	0.0794	0.6181	0.2753	0.7983	0.8527	0.6307	0.4131

SAE 8-6	Specificity	0.9813	0.4468	0.9894	0.8165	0.9826	0.9048	0.8397	0.7300
	Sensitivity	0.4262	0.7603	0.7297	0.7627	0.7358	0.9841	0.8333	0.9412
	*F*-score	0.5843	0.7699	0.8372	0.7258	0.8298	0.9185	0.4255	0.8050
	MCC	0.5259	0.2034	0.7608	0.5677	0.7773	0.8696	0.4268	0.6612

**Table 9:** Comparative experiment results.

Related researches	Test method	Classification accuracy (%)	Training set
Shahbaba and Neal [35]	5-fold CV	87.70	80%
Psorakis et al. [36]	10-fold CV	89.47	90%
Guo et al. [37]	10-fold CV	93.10	90%
Ozcift and Gulten [38]	10-fold CV	87.10	90%
Daliri [39]	50-50% training-testing	91.20	**50%**
Polat [40]	50-50% training-testing	97.93	**50%**
Chen et al. [41]	10-fold CV	96.07	90%
Zuo et al. [42]	10-fold CV	97.47	90%
Rao et al. [23]	10-fold CV	99.49	90%
This study	50-50% training-testing	94.00–98.00	**50%**

## References

[1] Marigliani C., Gates S., Jacks D., Morris M. S., Iansek R. N. (1997). Speech Pathology and Parkinson's Disease. *Parkinson's Disease: A Team Approach*.

[2] Miller N., Noble E., Jones D., Burn D. (2006). Life with communication changes in Parkinson's disease. *Age and Ageing*.

[3] Wooten G. F., Currie L. J., Bovbjerg V. E., Lee J. K., Patrie J. (2004). Are men at greater risk for Parkinsons disease than women?. *Journal of Neurology, Neurosurgery & Psychiatry*.

[4] Savitt J. M., Dawson V. L., Dawson T. M. (2006). Diagnosis and treatment of Parkinson disease: molecules to medicine. *Journal of Clinical Investigation*.

[5] Wang Y., Xie C.-L., Lu L., Fu D.-L., Zheng G.-Q. (2012). Chinese herbal medicine paratherapy for Parkinson's disease: a meta-analysis of 19 randomized controlled trials. *Evidence-based Complementary and Alternative Medicine*.

[6] Pan W., Kwak S., Liu Y. (2011). Traditional chinese medicine improves activities of daily living in parkinson's disease. *Parkinson's Disease*.

[7] Lees A. J. (1981). Early diagnosis of Parkinson's disease. *British Journal of Hospital Medicine*.

[8] Breen D. P., Evans J. R., Farrell K., Brayne C., Barker R. A. (2013). Determinants of delayed diagnosis in Parkinson's disease. *Journal of Neurology*.

[9] Cassani E., Barichella M., Cilia R. (2015). Simple and low-cost mucuna pruriens preparation for Parkinson’s disease patients in low-income countries. *Journal of the Neurological Sciences*.

[10] Rizzo G., Copetti M., Arcuti S., Martino D., Fontana A., Logroscino G. (2016). Accuracy of clinical diagnosis of Parkinson disease. *Neurology*.

[11] Jankovic J. (2008). Parkinson's disease: clinical features and diagnosis. *Journal of Neurology, Neurosurgery and Psychiatry*.

[12] Tolosa E., Wenning G., Poewe W. (2006). The diagnosis of Parkinson's disease. *Lancet Neurology*.

[13] Hughes A. J., Ben-Shlomo Y., Daniel S. E., Lees A. J. (1992). What features improve the accuracy of clinical diagnosis in Parkinson's disease: a clinicopathologic study. *Neurology*.

[14] Emre M., Aarsland D., Brown R. (2007). Clinical diagnostic criteria for dementia associated with Parkinson's disease. *Movement Disorders*.

[15] Ozkan H. (2016). A comparison of classification methods for telediagnosis of Parkinson's disease. *Entropy*.

[16] Esteva A., Kuprel B., Novoa R. A. (2017). Dermatologist-level classification of skin cancer with deep neural networks. *Nature*.

[17] Sewall G. K., Jiang J., Ford C. N. (2006). Clinical evaluation of Parkinson's-related dysphonia. *Laryngoscope*.

[18] Schley W. S., Fenton E., Niimi S. (1982). Vocal symptoms in parkinson disease treated with levodopa: a case report. *Annals of Otology, Rhinology & Laryngology*.

[19] De Brébisson A., Montana G. Deep neural networks for anatomical brain segmentation.

[20] Zhang M.-L., Zhou Z.-H. (2007). ML-KNN: a lazy learning approach to multi-label learning. *Pattern Recognition*.

[21] Sakar B. E., Isenkul M. E., Sakar C. O. (2013). Collection and analysis of a Parkinson speech dataset with multiple types of sound recordings. *IEEE Journal of Biomedical and Health Informatics*.

[22] http://archive.ics.uci.edu/ml/datasets/

[23] Rao S. S., Hofmann L. A., Shakil A. (2006). Parkinson's disease: diagnosis and treatment. *American Family Physician*.

[24] Jankovic J., Aguilar L. G. (2008). Current approaches to the treatment of Parkinson's disease. *Neuropsychiatric Disease and Treatment*.

[25] Varanese S., Birnbaum Z., Rossi R., Di Rocco A. (2010). Treatment of advanced Parkinson's disease. *Parkinson's Disease*.

[26] Yitayeh A., Teshome A. (2016). The effectiveness of physiotherapy treatment on balance dysfunction and postural instability in persons with Parkinson’s disease: a systematic review and meta-analysis. *Sports Medicine, Arthroscopy, Rehabilitation, Therapy & Technology*.

[27] Hartelins L., Svensson P. (1994). Speech and swallowing symptoms associated with parkinson’s disease and multiple sclerosis: a survey. *Folia Phoniatrica et Logopaedica*.

[28] Oxtoby M. (1982). *Parkinson’s Disease People and Their Social Needs*.

[29] Cools R. A., Jh D. V., Horstink W. M. (1990). Parkinson's disease: a reduced ability to shift to a new grouping if not prompted or guided. *Movement Disorders*.

[30] Rusz J., Cmejla R., Tykalova T. (2013). Imprecise vowel articulation as a potential early marker of Parkinson's disease: effect of speaking task. *Journal of the Acoustical Society of America*.

[31] Murdoch B., Whitehill T., De Letter M., Jones H. (2011). Communication impairments in parkinson's disease. *Parkinson's Disease*.

[32] Blin O., Desnuelle C., Rascol O. (1994). Mitochondrial respiratory failure in skeletal muscle from patients with Parkinson's disease and multiple system atrophy. *Journal of the Neurological Sciences*.

[33] Salenius S., Avikainen S., Kaakkola S., Hari R., Brown P. (2002). Defective cortical drive to muscle in Parkinson's disease and its improvement with levodopa. *Brain*.

[34] Fontana G. A., Pantaleo T., Lavorini F., Benvenuti F., Gangemi S. (1998). Defective motor control of coughing in Parkinson's disease. *American Journal of Respiratory and Critical Care Medicine*.

[35] Shahbaba B., Neal R. (2009). Nonlinear models using Dirichlet process mixtures. *Journal of Machine Learning Research*.

[36] Psorakis I., Damoulas T., Girolami M. A. (2010). Multiclass relevance vector machines: sparsity and accuracy. *IEEE Transactions on Neural Networks*.

[37] Guo P.-F., Bhattacharya P., Kharma N. (2010). Advances in detecting Parkinson’s disease. *Medical Biometrics*.

[38] Ozcift A., Gulten A. (2011). Classifier ensemble construction with rotation forest to improve medical diagnosis performance of machine learning algorithms. *Computer Methods and Programs in Biomedicine*.

[39] Daliri M. R. (2013). Chi-square distance kernel of the gaits for the diagnosis of Parkinson's disease. *Biomedical Signal Processing and Control*.

[40] Polat K. (2012). Classification of Parkinson's disease using feature weighting method on the basis of fuzzy C-means clustering. *International Journal of Systems Science*.

[41] Chen H.-L., Huang C.-C., Yu X.-G. (2013). An efficient diagnosis system for detection of Parkinson's disease using fuzzy *k*-nearest neighbor approach. *Expert Systems with Applications*.

[42] Zuo W.-L., Wang Z.-Y., Liu T., Chen H.-L. (2013). Effective detection of Parkinson's disease using an adaptive fuzzy *k*-nearest neighbor approach. *Biomedical Signal Processing and Control*.

[43] Shahbakhi M., Far D. T., Tahami E. (2014). Speech analysis for diagnosis of parkinson’s disease using genetic algorithm and support vector machine. *Journal of Biomedical Science and Engineering*.

[44] Little M. A., McSharry P. E., Hunter E. J., Spielman J., Ramig L. O. (2009). Suitability of dysphonia measurements for telemonitoring of Parkinson's disease. *IEEE Transactions on Biomedical Engineering*.

[45] Sakar C. O., Kursun O. (2010). Telediagnosis of parkinson's disease using measurements of dysphonia. *Journal of Medical Systems*.

[46] Little M. A., McSharry P. E., Roberts S. J., Costello D. A. E., Moroz I. M. (2007). Exploiting nonlinear recurrence and fractal scaling properties for voice disorder detection. *BioMedical Engineering Online*.

[47] Wang Y., Yao H., Zhao S. (2016). Auto-encoder based dimensionality reduction. *Neurocomputing*.

[48] Suk H.-I., Lee S.-W., Shen D. (2015). Latent feature representation with stacked auto-encoder for AD/MCI diagnosis. *Brain Structure and Function*.

[49] Huang Y., Li Y. (2004). Prediction of protein subcellular locations using fuzzy k-NN method. *Bioinformatics*.

[50] Ma C., Ouyang J., Chen H.-L., Zhao X.-H. (2014). An efficient diagnosis system for Parkinson's disease using kernel-based extreme learning machine with subtractive clustering features weighting approach. *Computational and Mathematical Methods in Medicine*.

